# Arylated analogues of cypronazole: fungicidal effect and activity on human fibroblasts. Docking analysis and molecular dynamics simulations

**DOI:** 10.1007/s13659-022-00329-0

**Published:** 2022-03-09

**Authors:** Natividad Herrera Cano, Sebastian A. Andujar, Cristina Theoduloz, Daniel A. Wunderlin, Ana N. Santiago, Guillermo Schmeda-Hirschmann, Ricardo D. Enriz, Gabriela E. Feresin

**Affiliations:** 1 Instituto de Biotecnología, Facultad de Ingeniería, Universidad Nacional de San Juan, CONICET–CCT San Juan, Av. Libertador General San Martín1109 (O), 5400 San Juan, Argentina; 2 Facultad de Química, Bioquímica y Farmacia- IMIBIO-SL (CONICET), Universidad Nacional de San Luis, Chacabuco 915, 5700 San Luis, Argentina; 3 Laboratorio de Cultivo Celular, Facultad de Ciencias de la Salud, Universidad de Talca, Casilla 747, 3460000 Talca, Chile; 4 ICYTAC, CONICET and Universidad Nacional de Córdoba, Facultad de Ciencias Químicas, Departamento, Química Orgánica, Ciudad Universitaria, Bv. Juan Filloy s/n, 5000 Córdoba, Argentina; 5 INFIQC, CONICET and Universidad Nacional de Córdoba, Facultad de Ciencias Químicas, Departamento Química Orgánica, Ciudad Universitaria, Haya de La Torre S/N, 5000 Córdoba, Argentina; 6 Laboratorio de Química de Productos Naturales, Instituto de Química de Recursos Naturales, Universidad de Talca, Casilla 747, 3460000 Talca, Chile

**Keywords:** Azoles, Antifungal cytotoxicity, Conformational and electronic analysis

## Abstract

Triadimefon (TDM) and cyproconazole (CPZ) are two triazoles widely used as fungicides. Several azoles were synthesised starting from commercial TDM and CPZ. The compounds were evaluated against phytopathogenic filamentous fungi, including *Aspergillus fumigatus* (AF), *A. niger* (AN), *A. ustus (AU), A. japonicus* (AJ), *A. terreus (AT), Fusarium oxysporum* and *Botrytis cinerea* isolated from grapevine in the province of San Juan, Argentina. Three of the synthesised compounds (1-(Biphenyl-4-yloxy)-3,3-dimethyl-1-(1H-1,2,4-triazol-1-yl)butan-2-one, **1**; 2-(Biphenyl-4-yl)-3-cyclopropyl-1-(1H-1,2,4-triazol-1-yl)butan-2-ol, **3**; 3-Cyclopropyl-2-(4’-fluorobiphenyl-4-yl)-1-(1H-1,2,4-triazol1-yl)butan-2-ol, **4**) presented remarkable in vitro fungicidal properties*,* with better effects than TDM and CPZ on some of the target fungi. Cytotoxicity was assessed using human lung fibroblasts MRC5. Derivative **1**, with IC_50_ values of 389.4 µM, was less toxic towards MRC-5 human lung fibroblasts than commercial TDM (248.5 µM) and CPZ (267.4 µM). Docking analysis and molecular dynamics simulations suggest that the compounds present the same interaction in the binding pocket of the CYP51B enzyme and with the same amino acids as CPZ. The derivatives investigated could be considered broad-spectrum but with some selectivity towards imperfect fungi.

**Graphical abstract:**

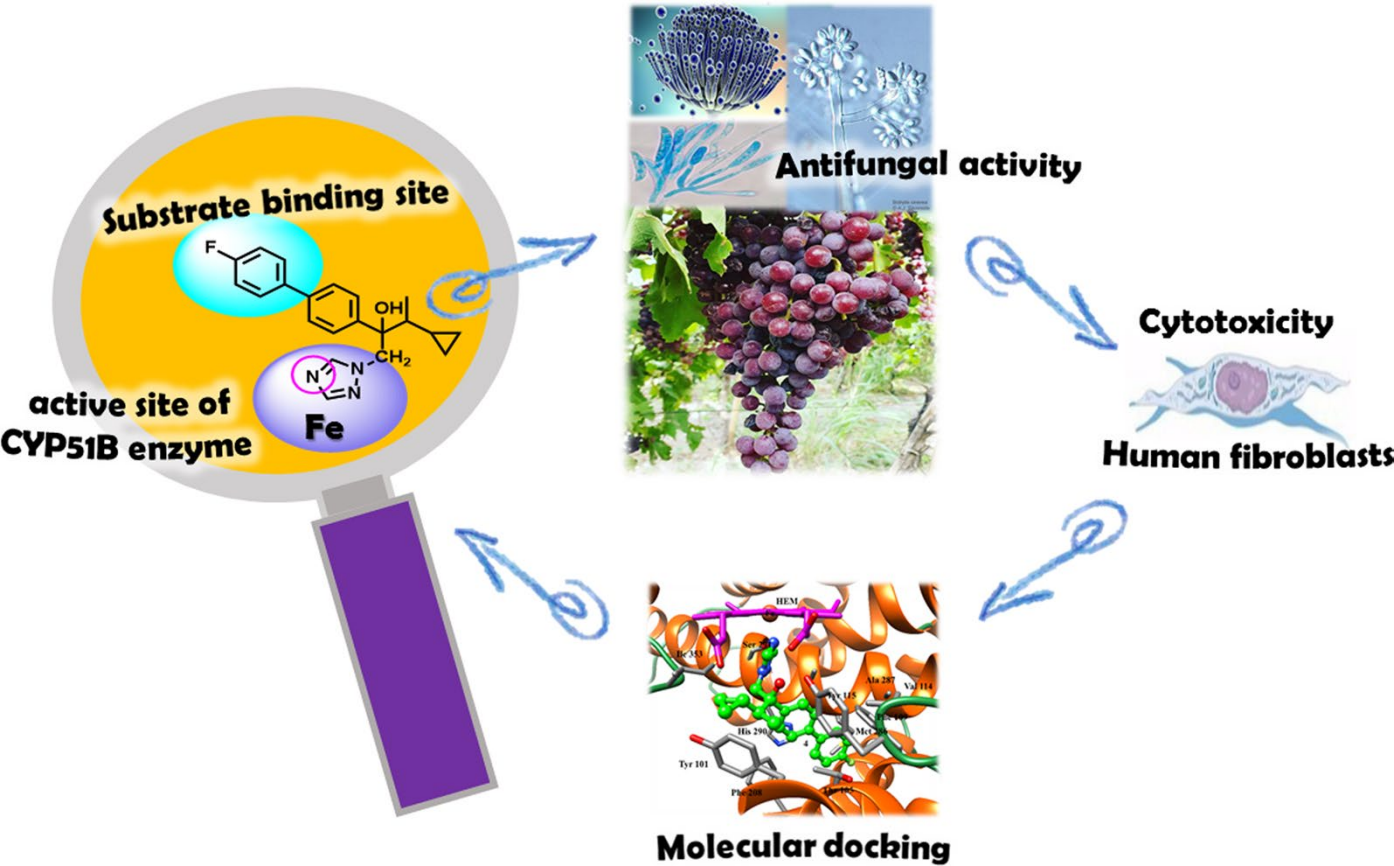

**Supplementary Information:**

The online version contains supplementary material available at 10.1007/s13659-022-00329-0.

## Introduction

Table and wine grapes (*Vitis vinifera L*.) as well as other relevant crops, including tomatoes (*Solanum lycopersicum*), apples, pears and citrus fruits, are very susceptible to a large number of pathogenic microorganisms such as *Botrytis cinerea*, *Aspergillus* spp., and *Fusarium* spp., among others. Fungal diseases cause economic losses during pre- and/or post-harvest, affecting production, processing and export, together with the quality of fruit. Argentina is the sixth wine producer worldwide [1], with the highest wine quality coming from the provinces of Mendoza, San Juan and Salta [2]. The grapes infected with moulds suffer alterations in the chemical composition, negatively affecting the flavour and colour of the wine and producing economic losses in the wine industry. The imperfect fungus *B. cinerea* is the causal agent of grey mould [3, 4]. It poses a major problem for vegetables and fruits during cold storage because the fungus can grow at low temperatures [5]. It is genetically variable and has developed strains resistant to many of the chemicals frequently used for its control [6].

The potential presence of mycotoxins in wine requires attention since countries with climatic conditions similar to those of Argentina and Uruguay have been reported to favour the development of ochratoxin A-producing fungi [7]. This is the only toxic substance of microbiological origin in wine for which there are specific international laws and regulations [8].

It is foreseen that the fungal resistance will increase with climatic change and higher temperatures. New environmentally friendly compounds with high effectiveness against phytopathogenic fungi and low toxicity will be needed to cope with the new challenges of food production.

**Fig. 1:**
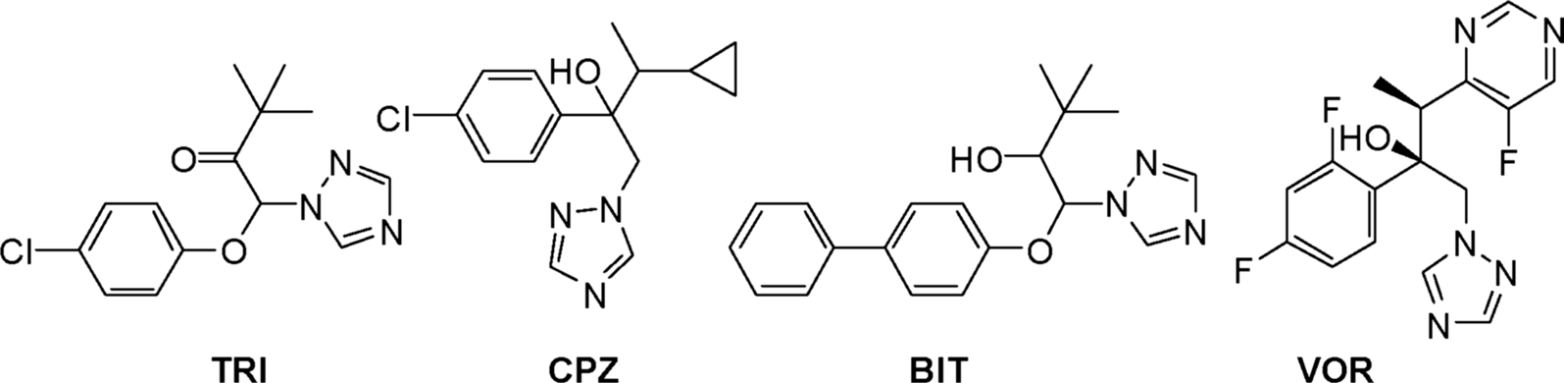
Chemical structures of triadimefon (TDM), cyproconazol (CPZ), bitertanol (BIT) and voriconazole (VOR)

About seventy percent of the commercial pesticides contain a heterocycle [9, 10]. Among them, triazoles are widely used in agriculture due to their known antifungal properties [11]. The triazoles 1-(4-chlorofenoxy)-3,3-dimethyl-1-(1H- 1,2,4-triazol-1-yl)butan-2-one (triadimefon®) (TDM) and 1-(4-chlorophenyl)-3-cyclopropyl-2-(1H-1,2,4-triazol-1-yl)butan-1-ol (cyproconazole®) (CPZ) (Fig. 1) are used to control *B. cinerea* in grapevine [12–14], but its use is widely questioned [15–17].

P450 14α-demethylase (CYP51) is the target enzyme of the triazoles derivatives. Voriconazole (VOR) and fluconazole (FLC**)** are two triazoles-derived antifungals used as models to study the mechanism of action. In 2015, Hargrove et al. reported X-ray data of voriconazole complexed to 14-α-demethylase of *A. fumigatus* (CYP51B), observing that the heterocyclic nitrogen atom N-4 binds to heme at the binding site of the enzyme in the fungus [18].

Besides, the fungi have developed mechanisms to avoid the action of azoles through mutations in the drug-binding site of Erg11p, reducing their binding affinity and generating resistance [19]. *Aspergillus* spp. contains two CYP51 isoenzymes, namely CYP51A and CYP51B. The resistance of *A. fumigatus* to FLC may be due to a point mutation in the amino acid sequence at the position corresponding to T 322 of CYP51A (threonine by isoleucine). However, CYP51B maintains the threonine, like most fungal species [20]. This information allows the analysis of the mechanism of action of structurally-related molecules, such as those reported in this work.

**Scheme 1:**
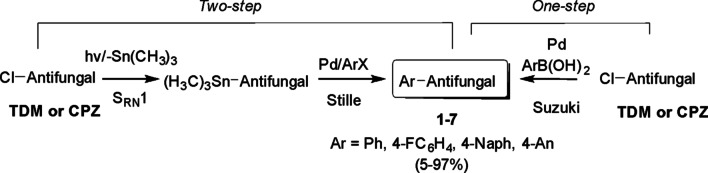
Methodologies employed for the arylation of TDM and CPZ

The synthesis of compounds **1**-**7** (Scheme 1) was accomplished by the arylation of TDM and CPZ (Fig. 1) via two different routes [21]. The synthetic strategy is shown in Scheme 1.

The first method involves a S_RN_1 [22, 23] substitution followed by the Stille reaction [24, 25]; while the second alternative consisted of a one-step reaction by the Suzuki coupling [26, 27].

The main goals of this work were: (a) to synthesise new fungicidal azoles according to Scheme 1; (b) to assess the antifungal activity and cytotoxicity of the azoles synthesised [21]; and (c) to conduct molecular modelling studies on the probable mechanism of action of the compounds.

## Results and discussion

### Synthesis of triadimefon and cyproconazole derivatives by Suzuki cross-coupling reactions catalysed by Pd

**Fig. 2:**
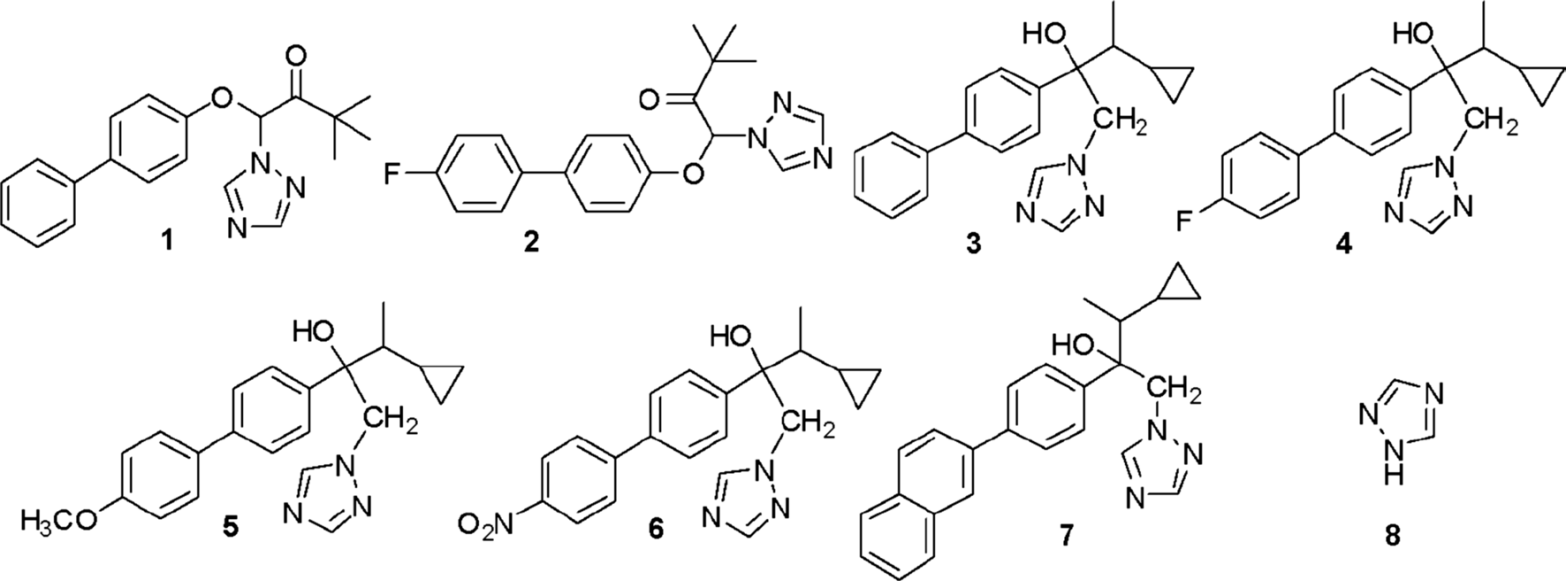
Chemical structures of compounds evaluated

Compounds **1**–**7** were synthesised by arylation from the commercial compounds TDM and CPZ (Fig. 2).

The synthetic strategy was to substitute the chlorine atom from commercial antifungals (TDM and CPZ) with other less toxic groups while maintaining the triazole moiety. This synthetic strategy was reported^17^ by two different synthetic alternatives, namely a sequence of S_RN_1-Stille coupling or the Suzuki coupling reaction (Scheme 1). The coupling of TDM and CPZ with different boronic acids (R = *p*-F; naphthalene, *o*-OCH_3_, *p*-OCH_3_, *p*-NO_2_) by the Suzuki reaction was studied using Pd/C as catalyst and dimethylacetamide/water (20:1) as solvent (Table 1, entries 1–5).

**Table 1:** Suzuki coupling of TDM and CPZ with different boronic acids and (2-biphenyl) dicyclohexylphosphine as ligand


Entry	Electrophile ArB(OH)_2_	% Yield of product from TDM^a^	% Yield of product from CPZ^a^	References
1		**1** (100)^b^	**3** (97)^b^	Cano et al. [21]
2		**2** (100)^b^	**4** (97)^c^	Cano et al. [21]
3		−^d^	**5** (45)^c^	This work
4		−^d^	**6** (5)^c^	This work
5		(5)^e^	**7** (36)^c^	Cano et al. [21]

^a^Arylboronic acid (2 equiv) was added each 12 h. Four equivalents of arylboronic acid were used, except for 4-fluorophenylboronic acid (2 equivalents used)

^b^Organic products were determined by GC using relative areas

^c^Isolated yields

^d^The reaction was not carried out

^e^Non-isolated product

The reactions afforded a series of TDM and CPZ derivatives with poor to good yields (5–100%), depending on the electrophile. The best yields were obtained using phenylboronic acid and 4-fluorophenylboronic acid with less steric hindrance (Table 1, entries 1 and 2). Furthermore, the poor reactivity observed with naphthylboronic acid is probably due to steric hindrance (Table 1, entry 5).

The different reactivity observed with 4-methoxyphenylboronic (45%) and 4-nitrophenylboronic (5%) can be explained by the effect of ring substituents (Table 1, entries 3 and 4). It is well known that boronic acids with electron withdrawing substituents in the ring reduce the transmetalation step in the catalytic cycle, decreasing the reaction yield [17]. Then, all new synthesised compounds were assessed for fungicidal and cytotoxicity effects.

### Antifungal activity in vitro Assay

Fungal growth was assessed at six concentrations (3.1–100 μg/mL), using enriched Czapek broth in the presence of triazole **1-8** (Fig. 2). Each treatment was carried out in triplicate. The Minimum Inhibitory Concentration (MIC_100_) was determined and was defined as the minimum concentration required to inhibit the complete fungal growth inhibition under the conditions of the assay. Triadimefon (TDM) and cyproconazole (CPZ) were used as positive control. In order to evaluate the efficacy of the new compounds to control fungi affecting vineyards, imperfect fungi isolated from infected grapes from the province of San Juan in Argentina were employed. Results are shown in Table 2. Significant differences were determined by *t*-Student hypothesis test *p*, at 95% confidence. In general, moderate to good activity was observed compared to the reference compounds as well as some selectivity against microorganisms.

**Table 2:** Log P values, fungicidal activity (MIC_100_ values in µg/mL) and cytotoxicity towards human lung fibroblasts MRC5 (IC_50_ values in µM) for compounds **1**–**8** and reference compounds

Entry	Compound	*Log P*^a^	Fungicidal activity MIC_100_ (µg/mL)							*Cytotoxicity* IC_50_ (µM ± SD)
			BC	AF	AJ	AN	AT	AU	F	
**1**	**1**	3.92	10 ± 0.7	80 ± 2.5	≥ 100	50 ± 1.7	≥ 100	50 ± 1.2	≥ 100	389.4 ± 23.5
**2**	**2**	4.09	≥ 100	≥ 100	≥ 100	≥ 100	≥ 100	≥ 100	≥ 100	90.0 ± 4.3
**3**	**3**	3.80	25 ± 1.4	≥ 100	12.5 ± 0.8	3.12 ± 0.3	≥ 100	6.25 ± 0.3	5 ± 0.4	118.5 ± 9.4
**4**	**4**	3.95	12.5 ± 0.9	12.5 ± 0.8	15 ± 0.7	10 ± 0.5	10 ± 0.4	14 ± 0.7	≥ 100	61.1 ± 3.7
**5**	**5**	3.72	≥ 100	≥ 100	≥ 100	6.25 ± 0.4	≥ 100	≥ 100	≥ 100	732.1 ± 29.3
**6**	**6**	3.55	≥ 100	≥ 100	≥ 100	≥ 100	≥ 100	≥ 100	≥ 100	ND
**7**	**7**	4.98	≥ 100	≥ 100	≥ 100	≥ 100	≥ 100	6.25 ± 0.3	≥ 100	49.1 ± 3.4
**8**	**8**	−0.28	≥ 100	≥ 100	≥ 100	≥ 100	≥ 100	≥ 100	≥ 100	> 1000
**9**	TDM	2.89	80 ± 2.2	80 ± 2.4	≥ 100	≥ 100	≥ 100	80 ± 2.6	≥ 100	248.5 ± 12.4
**10**	CPZ	2.63	12.5 ± 0.8	25 ± 1.4	25 ± 1.3	25 ± 1.5	15 ± 0.9	15 ± 0.7	≥ 100	267.4 ± 18.7
**11**	VOR	0.52	˂ 0.75	˂ 0.75	˂ 0.75	1.25 ± 0.4	6.25 ± 0.8	1.25 ± 0.5	˂ 0.75	ND

BC, *B. cinerea* (isolated from grapes); AF, *Aspergillus fumigatus* (ATTC 26934); AJ, *A. japonicus* (M15C); AN, *A. niger* (ATCC 9029); AT, *A. terreus* (M16C); AU, *A. ustus* (PN-S4); F, *Fusarium oxysporum* (M15-Pa). All strains were obtained from the culture collection of Instituto de Biotecnología, Universidad Nacional de San Juan (IBT). ND, Not determined. The log P values were estimated using ChemBioDraw Ultra 12.0^a^

Fungus *B. cinerea* was more sensitive to compounds **1, 4** and **3**, with MIC_100_ values of 10 µg/mL (29.8 µM), 12.5 µg/mL (35.5 µM) and 25 µg/mL (75.0 µM), respectively. Compound **4** showed a similar effect to the reference drug CPZ. These compounds were more effective against *B. cinerea* than TDM (MIC = 80 µg/mL, 274.0 µM). Compound **1** is the oxidised (keto) form of bitertanol (BIT) (Fig. 1), which is a known fungicide used to control certain diseases in fruits and vegetables [28]. Compounds **5**-**8** did not show fungicidal activity against *B. cinerea*. Molecular modelling studies demonstrated that compounds **1**, **3** and **4** interacted with the catalytic site in a similar way than CPZ and VOR. The inactive compounds **5**-**8** had considerably weaker interactions (see discussion in the “Molecular Modelling” section).

Concerning *Aspergillus* sp., they showed only moderate fungicidal effects. Thus, derivative **4** (MIC_100_ = 12.5 µg/mL, Table 2, entry 4) stands out against *A. fumigatus* as it significantly improved the inhibition produced with respect to the controls TDM and CPZ (MIC_100_ = 80 µg/mL and 25 µg/mL, respectively; Table 2, entry 9 and 10). This last compound becomes a valuable candidate because it was the only one showing improvements in potency against *A. fumigatus* when compared with other synthesised compounds.

The best candidates selected against *A. japonicus* were **3** and **4,** which proved to be comparable to TDM and CPZ (MIC_100_ = 12.5 µg/mL and 15.0 µg/mL; Table 2, entries 3 and 4).

For *A. niger*, azoles **3, 4** and **5** have shown powerful fungicidal activity with MIC_100_ values of 3.12, 10 and 6.25 µg/mL, respectively (Table 2, entries 3, 4 and 5). Whereas for derivative **4,** a selective effect was detected against *A. terreus*, (MIC_100_ = 10.0 µg/mL, overcoming the inhibition observed for the controls TDM and CPZ (> 100 µg/mL, and 15.0 µg/mL respectively).

**Fig. 3:**
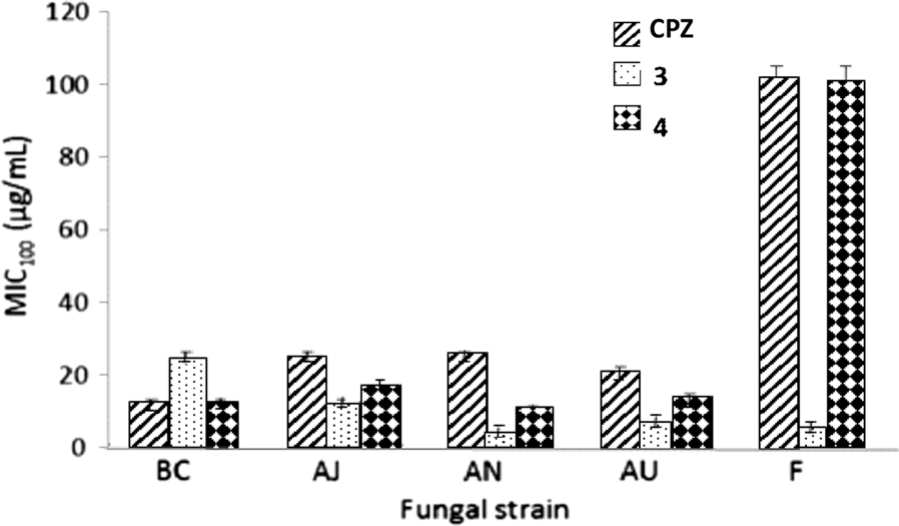
Antifungal activity of compounds **3**, **4** and CPZ against filamentous fungi. Fungal strains: BC*: Botrytis cinerea*; AJ: *Aspergillus japonicus*; AN: *Aspergillus niger*; AU: *Aspergillus ustus*; F: *Fusarium oxysporum*

Moreover, **3, 4** and **7** displayed outstanding activity against *A. ustus* (MIC_100_ = 6.25 µg/mL for **3** and **7,** and 14.0 µg/mL for **4**), higher than that detected with TDM and CPZ. These MIC_100_ were similar to those observed for the reference compound to VOR (MIC_100_ = 1.25 µg/mL). The change of chlorine (CPZ) to phenyl (**3**) or fluorophenyl (**4**) in the aromatic ring might account for the enhanced activity observed with **3** and **4** (Fig. 3). This improved activity could be explained in terms of an increased lipophilicity in both compounds.

On the other hand, for *F. oxysporum*, the derivatives tested did not cause growth inhibition of the fungus, except for derivative **3**, which presented a MIC_100_ value equal to 5 µg/mL. Controls were ineffective against this strain, highlighting the importance of this derivative in the control of this problematic pest.

According to the results obtained in the antifungal susceptibility tests, it is possible to classify the compounds based on their action as broad-spectrum or reduced-spectrum fungicides. Among the compounds that showed broad activity, derivative **4** showed a remarkable inhibitory effect against all the microorganisms tested, except for *F. oxysporum*. In addition, derivatives **1** and **3** also presented significant activity against most of the strains studied. This last compound, unlike its fluorinated analogue **3**, presented an inhibitory effect against *F. oxysporum.* Derivatives **5** and **7** are also worthy of interest because they allowed the selective growth of *B. cinerea*, *F. oxysporum* and of some strains of *Aspergillus,* reaching an adequate inhibition against *A. niger* (**5**) and *A. ustus* (**7**) (reduced spectrum antifungals).

The metabolism of certain species of filamentous fungi, such as *A. niger* and *A. ustus*, produces bioactive compounds against other microorganisms and are used as biocontrol agents. As a result, they could be combined with derivatives **5** or **7** to design more effective therapies for the control of pest resistant to conventional therapies, enhancing efficiency and reducing the impact on the environment.

On the other hand, the cytotoxicity of TDM, CPZ, **1**–**5**, **7** and **8** was assessed on MRC-5 cells by the neutral red uptake method and results are summarised in Table 2. Considering the broad-spectrum (**1**, **3** and **4**), low to high cytotoxicity values were observed (MIC_50_: 389.4, 118.5 and 61.1 µM, respectively). In this way, derivative **1** presented the lowest levels of cytotoxicity, even lower than those of the TDM and CPZ controls. Derivative **3** presented moderate cytotoxicity, comparable to that of commercial antifungals, but its activity against certain strains was considerably higher. In addition, the most active compound in the series (**4**) presented high cytotoxicity but given that it causes the same effect at a lower dose than the controls, its toxicity would be comparable to that of TDM and CPZ. These compounds could be suitable candidates to replace commercial ones, preventing the emergence of resistance phenomena in other species.

When the reduced-spectrum compounds were analysed, derivative **5** presented low cytotoxicity (MIC_50_ = 732.1 µM), while derivative **7** had the highest toxicity in the series with a value of MIC_50_ = 49.1 µM, in coincidence with its lower polarity (log P = 4.98) when compared to other derivatives. This highlights the importance of derivative **5** as a specific fungicidal agent against *A. niger* and as an alternative in the design of a combination therapy of the compound with *A. terreus*.

Compound **8** had a higher hydrophilicity than the series investigated (log P =−0.2 value), was less cytotoxic and inactive as an antifungal under the experimental conditions assayed. Considering all these results, it is possible to affirm that the triazole ring did not show fungicidal activity by itself. Rosenkranz [29] reported that compounds with higher lipophilicity showed increased cellular toxicity. With a few exceptions, these current results exhibit a good correlation between both variables. Considering that simple structural modifications led to higher activity and lower cytotoxicity, selective modifications may produce promising new fungicidal azoles.

### Assay in greenhouse conditions

**Fig. 4:**
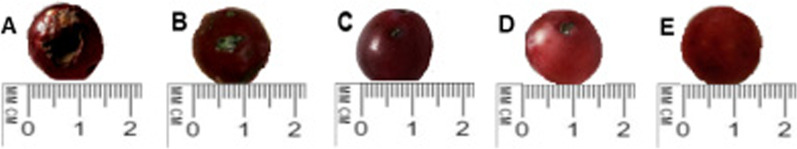
In vivo protection efficacy of compound **4** and cyproconazole against *B. cinerea*. **A** Blank control; **B** Positive control (cyproconazole, 2.5 μg/mL); **C** cyproconazole, 12.5 μg/mL; **D** **4**, 2.5 μg/mL; **E** **4**, 12.5 μg/mL

The potential usefulness of compound **4** against *B. cinerea* was assessed under greenhouse conditions using infected grapes (in vivo), at 7 days of exposure. The compound was evaluated at 2.5 μg/mL and 12.5 μg/mL, CPZ was used as positive control (at 12.5 μg/mL). Both compounds 4 and CPZ showed potent fungicidal activity at 12.5 μg/mL (CIM_100_), displaying an efficacy of 100% and 75.3%, respectively. At the concentration of 2.5 µg/mL, the efficacy was lower, with a preventive rate of 50.6% for **4** and 38.3% for CPZ, respectively. The blank control (untreated grapes) was completely affected by *B cinerea* (100% incidence) (Fig. 4).

### Effect of *B. cinerea* on hyphal morphology

**Fig. 5:**
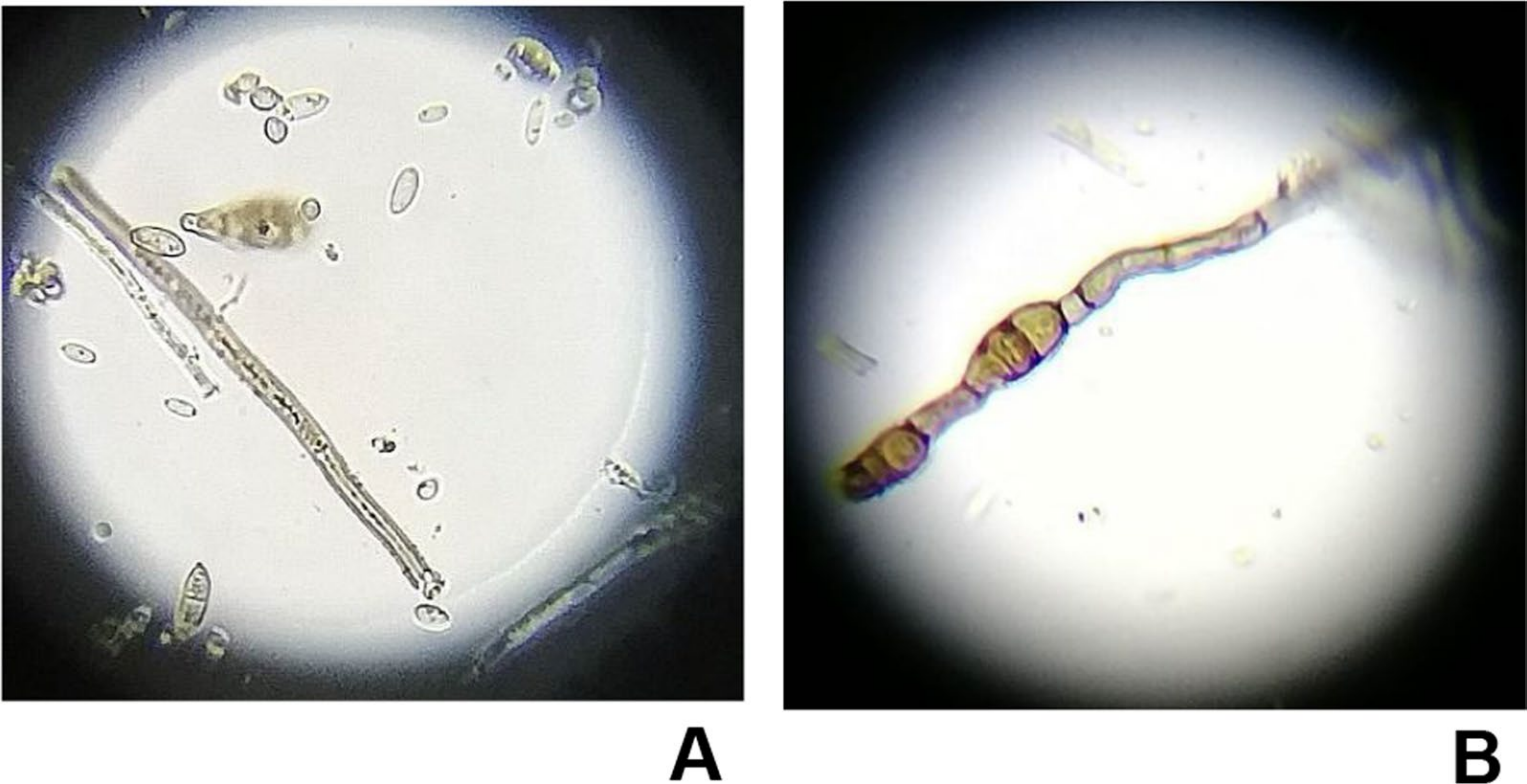
Microscopic observation of hyphal morphology of (**A**) *B. cinerea* from the blank control observing regular hyphae and (**B** cultures treated with compound **4** showing deformed mycelia with internal vacuoles of *B. cinerea*

Changes in the hyphae morphology of *B. cinerea* treated with compound **4** were observed using optical microscopy. Homogeneous hyphae of linear morphology and constant diameter were observed in the control (2% of DMSO) (Fig. 5). Micrographs of *B. cinerea* mycelium treated with derivative **4** (12.5 μg/mL) showed morphological alterations, including irregular hyphae, variable diameter and the presence of internal vacuoles, which were not observed in untreated mycelia (blank control). These results showed that *B. cinerea* is affected by compound **4**, which causes widespread damage, including cytosolic vacuoles, hyster disruption and hyphal wall lysis.

### Molecular modelling

**Fig. 6:**
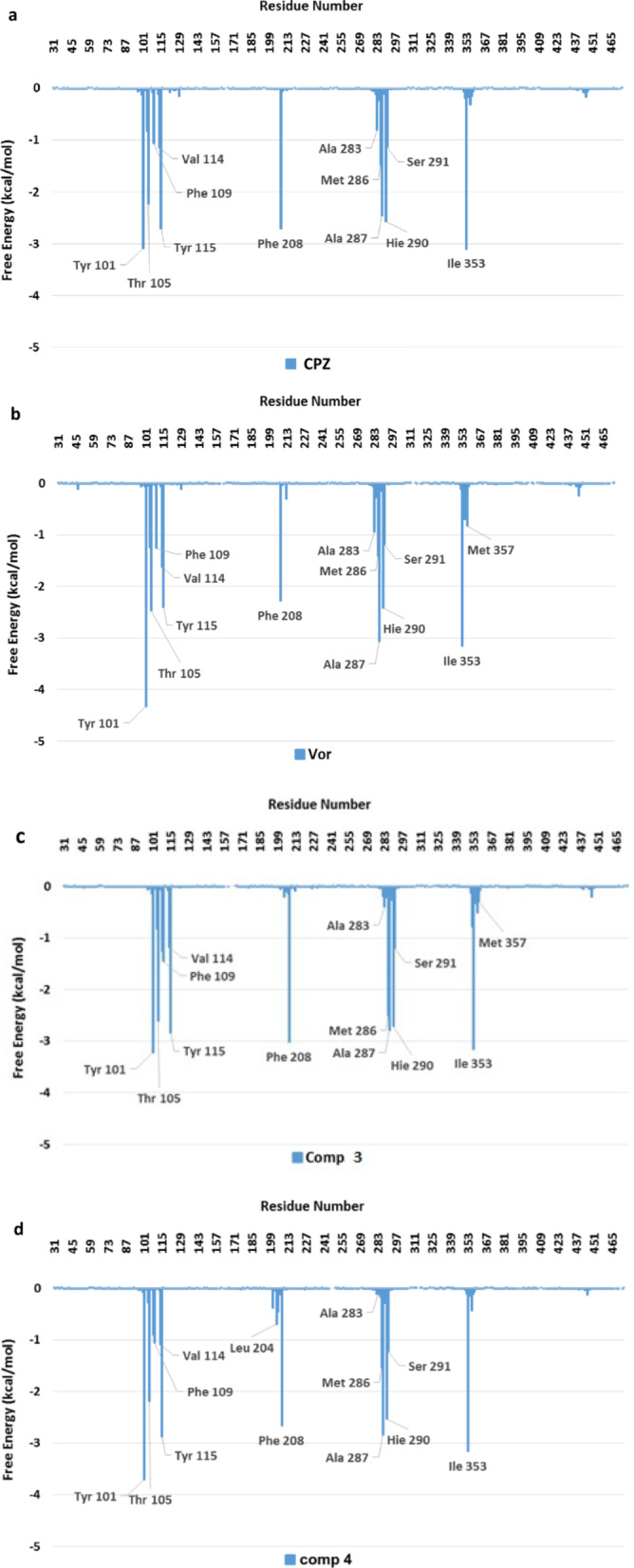
Histograms of interaction energies partitioned with respect to the amino acids of 14α-demethylase complexed with compounds: **a** CPZ, **b** VOR, **c** 3 and **d** 4. The x-axis denotes the residue number of 14α-demethylase, and the y-axis denotes the interaction energy between the compounds and specific residue. Negative values and positive values are favourable or unfavourable to binding, respectively

**Fig. 7:**
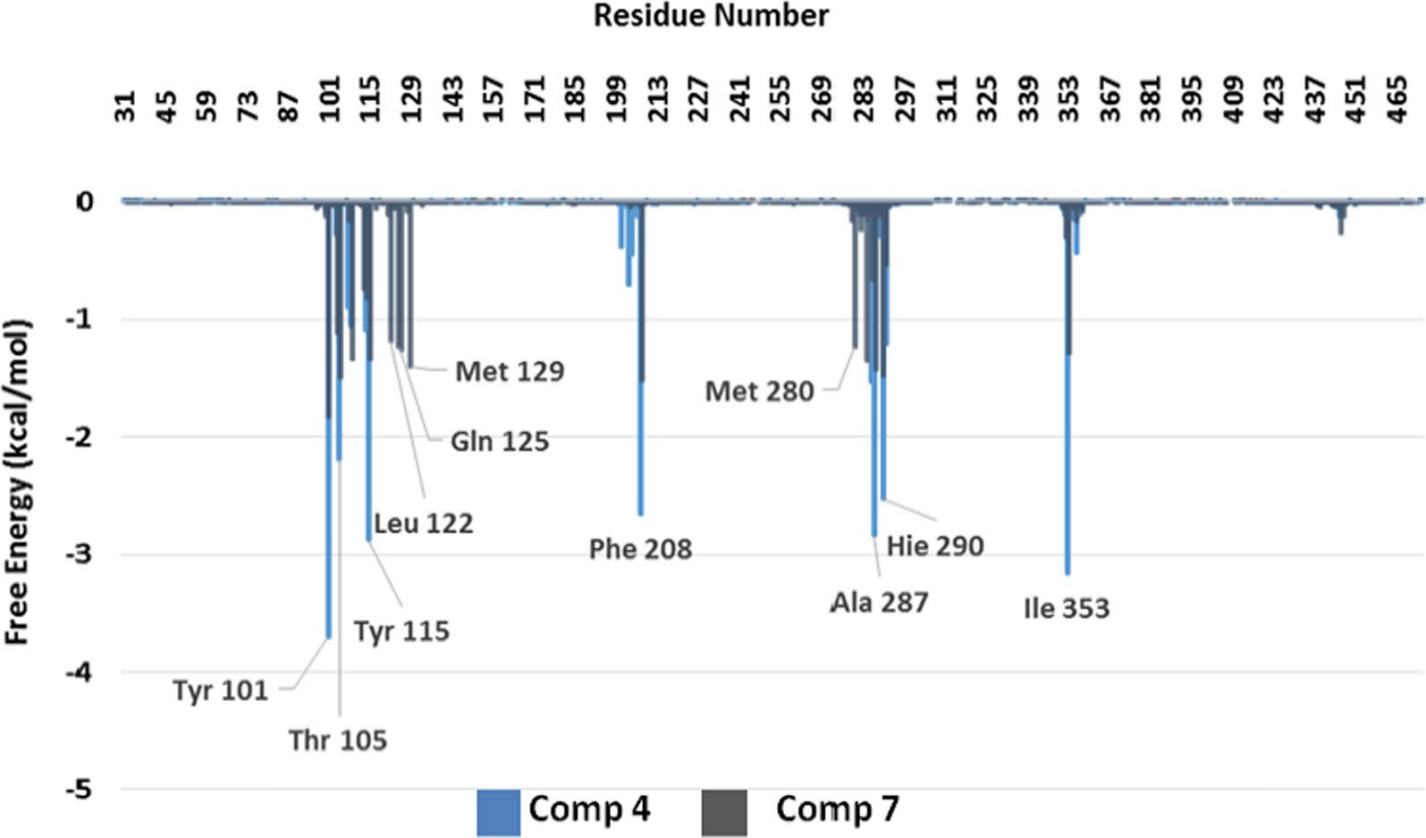
Interactions obtained for compounds **4** (light blue) and **7** (black) with the active site of the enzyme

A molecular modelling study was performed to explain the experimental results of the antifungal activity. The study was conducted in two steps. Docking experiments were carried out first, followed by molecular dynamics (MD) simulations of all the complexes. Since we lacked experimental data (X-ray crystallography) for the complexes with our ligands, we relied on simulation techniques and docking calculations. Docking programs present two main limitations: the conformational space reduced by imposing limitations on the system (a rigid receptor and fixed bond angles and lengths in the ligand) and the simplified scoring function, often based on empirical free energies of binding at each step of the conformational search. One approach to solving such limitations is to employ molecular dynamic and/or free energy perturbation simulations (ref. “Computational protein–ligand docking and virtual drug screening with the AutoDock suite” Nat Protoc. 2016 May; 11(5):905–19. https://doi.org/10.1038/nprot.2016.051). Therefore, we have complemented the docking study using MD simulations. From the MD simulations, an analysis per residue was performed using the MM-GBSA method for all the compounds reported. Figure 6 shows the histograms for compounds **3**, **4**, VOR and CPZ. This figure shows that compounds 3 and 4 have almost the same interactions than VOR and CPZ with the same amino acids in the catalytic site, with similar strengths in these interactions. Compounds **5**, **6** (Additional file 1: Figure S1) and **7** (Fig. 7) show much weaker interactions than compounds **3** and **4**.

These results suggest that the azoles investigated have similar interactions to those of VOR and CPZ in the active site of BcCYP51.

Figure 7 compares the interactions obtained for compounds **4** and **7** along with the simulations. Interactions of compound **7 **(black) are significantly weaker than the interactions obtained for compound **4** (light blue). Interactions obtained for compounds **5** and **6** are very similar to those observed for compound **7** (Additional file 1: Figure S1). These results are in full agreement with the experimental data, and explain why compounds **3** and **4** are more active than **5**-**7** against *B. cinerea*. Based on these findings, we can infer that the same explanation might be extended to *A. niger* and *A. terreus.*

**Fig. 8:**
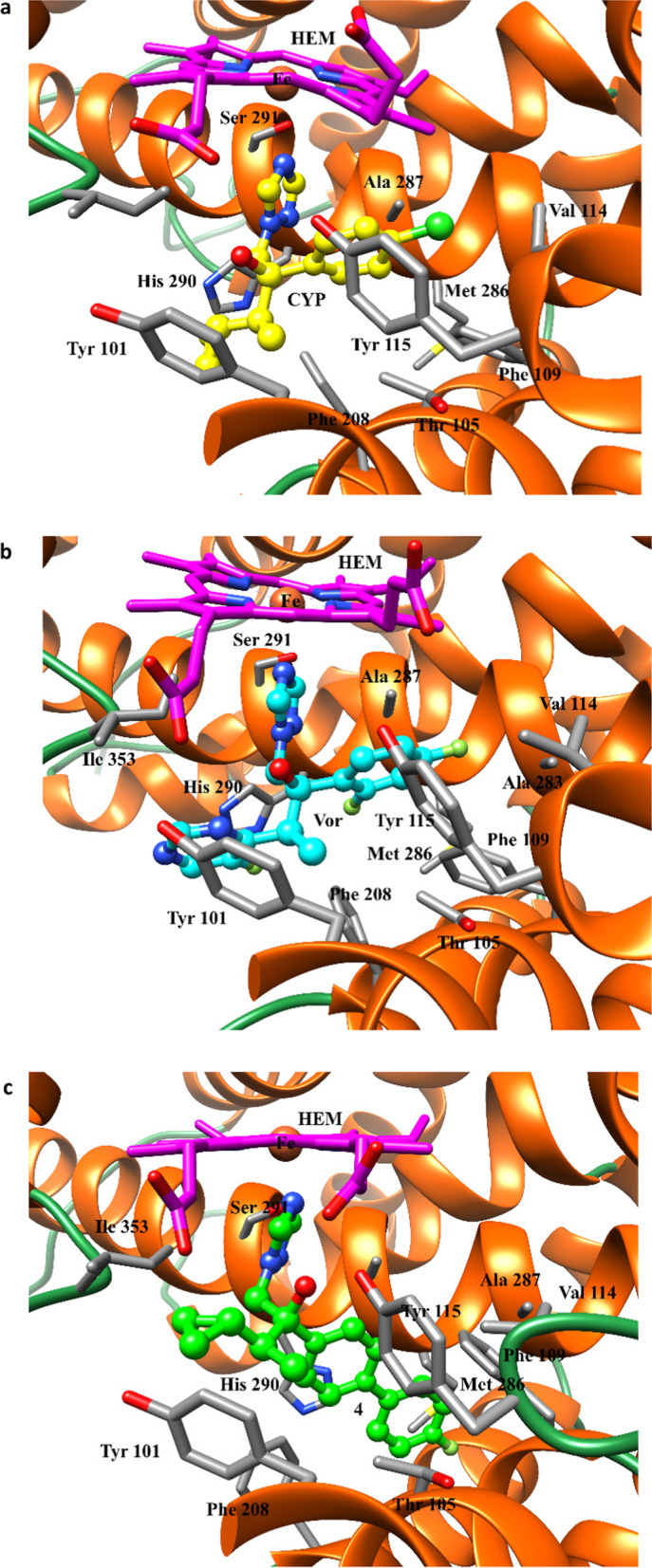
Spatial view of the different complexes with 14α-demethylase: **a** CPZ; **b** VOR, and **c** Compound **4**. The names of the main residues involved in the stabilizing interactions are shown in the figure

The different molecular interactions involved in the complexes’ stabilisation are better appreciated in Fig. 8, where a special view of the different complexes with each active compound is shown.

The main interactions take place between the triazole ring and the iron atom. There are significant interactions with Tyr101, Thr105, Phe109, Tyr115, Phe208, Met290, Ala287, His290 and Ile353, which stabilise these molecular complexes. It should be noted that these complexes are very similar and, therefore it is reasonable to infer that the compounds described herein have a mechanism of action closely related to that of CPZ and VOR.

The combination of experimental data, docking analysis and MD simulations showed that compounds **3** and **4** interact with the same binding pocket of the CYP51B enzyme and with similar amino acids as CPZ. However, the interactions obtained for compounds **5**, **6** and **7** are weak in accordance with the experimental bioassay data and provide additional support. Thus, these simulations offer a satisfactory explanation of different fungicidal activities observed for these new azole derivatives.

## Conclusions

The antifungal effect of synthetic triazoles related to CPZ and TDM against a panel of phytopathogenic filamentous fungi is of agricultural relevance. The new series of azole derivatives presented interesting fungicidal activity. Compounds **3** and **4** displayed strong activity against *A. japonicus*, *A. niger* and *A. ustus*, outperforming the commercial drug CPZ. Products **1**, **3** and **4** were more effective against *B. cinerea* than TDM. Derivative **4** effectively controlled the development of *B. cinerea* in infected grapes under greenhouse conditions, and it is a potential candidate against native *B. cinerea* strains from the province of San Juan, Argentina.

Fungal lanosterol 14α-demethylase belongs to the CYP51 class in the cytochrome P450 superfamily of enzymes. It is the key enzyme in the biosynthesis of ergosterol. The molecular modelling study provides a satisfactory explanation of the antifungal activity obtained against phytopathogenic fungi and suggests that the synthesised compounds interact with lanosterol 14α-demethylase. As a result, further research on other crops and phytopathogenic fungi is encouraged.

## Experimental section

### Materials and methods

#### Chemicals

Compounds 4-methoxyphenylboronic acid, 4-nitrophenyl boronic acid, Pd/C,[1,1'-biphenyl]-2-yldicyclo hexyl phosphine and K_2_CO_3_ were purchased from Sigma-Aldrich Chemical Co., and were used without further purification. CPZ, TDM and VOR were commercially available and used as received. Dimethylacetamide was purchased from Merck KGaA.

### General methods

^1^H and ^13^C NMR spectra were recorded on a Bruker Advance II 400 spectrometer, operating at 400 MHz for ^1^H and 100 MHz for ^13^C, respectively, using CDCl_3_ as solvent. Coupling constants (J) are given in Hz. GC analyses were performed on a GC/MS QP 5050 spectrometer equipped with a VF-5 ms. Ionization was achieved by electronic impact (70 eV) and detection setup in positive mode. Radial chromatography separations were performed on a Chromatotron 7924 T Model. Mass spectra were measured on a Bruker MicroTOF Q II equipment, operated with an ESI source in positive mode. Nitrogen was used as nebulising gas and drying gas, and 10 mM sodium formiate was used as internal calibration. Melting points were determined on a Digital Melting Point, Electrothermal IA9100, (Cole-Parmer), Staffordshire, ST15 OSA, UK apparatus and are uncorrected. The microscope used for the micrographs was the Ecoline binocular microscope model XSP-42.

### Pd-catalysed Suzuki cross-coupling reactions

In a Schlenk tube under an inert nitrogen atmosphere, Pd/C (0.025 mmol), 4-methoxyphenylboronic acid (1 mmol), K_2_CO_3_ (1 mmol) and ligand (0.011 mmol) were added under magnetic stirring. Then, dimethylacetamide (2.35 mL) in water (0.15 mL) was added into the tube. The solution was deoxygenated and refilled with nitrogen three times before adding TDM or CPZ (0.5 mmol). Compounds TDM or CPZ (0.5 mmol) were added to the reaction mixture, which was then heated to 80 ºC for 48 h. After completion of the reaction, the reaction mixture was cooled and stopped by the addition of 3 mL of water and was extracted with dichloromethane (3 × 10 mL). The organic layer was dried over Na_2_SO_4_ and concentrated in a vacuum. The combined organic extract was analysed by GC–MS. Products were isolated by radial chromatography, using ethyl acetate as the mobile phase.

### Compound description

The spectroscopy data of the compounds **1**-**4** and **7** is summarised in [21].

**3-Cyclopropyl-2-(4'-methoxy-[1,1'-biphenyl]-4-yl)-1-(1H-1,2,4-triazol-1-yl)butan-2-ol** (**5**). The product was isolated as a colourless oil after preparative thin layer chromatography using dichloromethane/ethyl acetate (75/25). ^1^H NMR (CDCl_3_, 400 MHz), *δ* (ppm): 0.070- 0.61 (m, 6 H); 1.02 (d, 3H, J = 9.67 Hz); 1.21–1.29 (m, 1H); 3.84 (s, 3H); 4.49 (d, 1 H, J = 13.96 Hz); 4.56 (s br, OH);4.90 (d, 1H, J = 13.96 Hz); 7.14–7.31 (m, 8H); 7.76 (s, 1 H); 7.85 (s, 1H). ^13^C NMR (CDCl_3_, 100 MHz), *δ* (ppm): 3.2; 6.6; 13.7; 14.7;15.2; 47.5; 48.0; 57.3; 58.3;79.6; 80.0;114.5; 127.0; 128.0; 128.1; 128.3; 128.7; 131.4; 131.8; 140.4;152.0, 158.2. MS (EI+) m/z(%): 296 (2),295 (23), 294 (100), 253 (5), 252 (10), 251 (8), 190 (7), 189 (37), 188 (18),176 (9),174 (10),161 (8),159 (9), 147 (10),146 (10),135 (7),134 (14),133 (19),105 (58),104 (13), 91 (22), 77 (19). HRMS [MH]^+^ exact mass calcd for C_22_H_26_N_3_O_2_: 364.2025, found: 364.20300.

**3-Cyclopropyl-2-(4'-nitro-[1,1'-biphenyl]-4-yl)-1-(1H-1,2,4-triazol-1-yl)butan-2-ol** (**6**). The product was isolated as a colourless oil after preparative thin layer chromatography using dichloromethane/ethyl acetate (50/50). ^1^H NMR (CDCl_3_, 400 MHz), *δ* (ppm): 0.85–1.37 (m, 5H); 1.58–1.71 (d, 3H, J = 9.47 Hz); 1.74–1.83 (m, 1H); 4.50 (d, 1H, J = 13.73 Hz); 4.57 (s br, OH); 4.91 (d, 1 H, J J = 13.73 Hz); 7.13–7.50 (m, 8H); 7.78 (s, 1H); 7.84 (s, 1H).^13^C NMR (CDCl_3_, 100 MHz), *δ* (ppm): 3.1; 3.2; 7.8; 13.1; 30.0; 57.3; 79.6; 113.2; 114.5; 127.0; 128.0; 128.1; 128.3; 128.7; 131.4; 131.8; 133.4; 140.4; 141.8; 152.0, 162.2. MS (EI^+^) m/z (%):312 (3), 311 (17), 310 (100), 264 (27), 257 (4), 256 (11), 198 (45), 82 (19), 77 (21). HRMS [M^+^Na]^+^ exact mass calcd for C_21_H_22_ NaN_4_O_3_: 401,1584, found: 401,1586.

**1H-1,2,4-Triazole** (**8**).^1^HNMR (400 MHz, CDCl_3_), *δ *(ppm): 8.32 (s, 2H), 13.42 (s, H) [30]. DMSO-d_6_ 147.0, 147.0.^35^ MS, m/z: 69 (M+); Anal calc. (%) for C_2_H_3_N_3_:C, 34.78; H, 4.38; N, 60.84; Found C, 29.63; H, 4.34; N, 60.62.

### Microorganisms

The imperfect fungi used for the assays included *Botrytis cinerea* isolated from grapevine samples, *Aspergillus fumigatus* (ATTC 26934), *A. niger* (ATCC 9029), *A. ustus* (PN-S4), *A. japonicus* (M15C), *A. terreus* (M16C) and *Fusarium oxysporum* (M15-Pa) isolated from soil samples from the province of San Juan, Argentina. The microorganisms were grown on Czapek-yeast extract agar (CYA) prepared with yeast extract, K_2_HPO_4_, saccharose, agar, distilled water and enriched with a solution of mineral salts (NaNO_3_, KCl, MgSO_4_.7H_2_O and FeSO_4_.7 H_2_O). Inocula were adjusted to 1–5 × 10^4^ colony forming units (CFU/ mL).

### Bioassays

#### In vitro antifungal activity

The minimum inhibitory concentration (MIC) of each compound was evaluated using broth microdilution techniques in 96-well microtiter plates according to the guidelines of the National Committee for Clinical Laboratory Standards for filamentous fungi M 38 A. The starting inoculum was 1–5 × 10^4^ CFU/mL in accordance with the Clinical and Laboratory Standards Institute (CLSI, formerly the National Committee for Clinical Laboratory Standards [NCCLS]) (M27-A3 document for yeasts, and M38-A2 document for filamentous fungi) [31].

Microtiter plates were incubated at 30 °C for *Aspergillus* and *Fusarium*, and 22 °C for *B. cinerea* in a moist and dark chamber. MIC values were recorded after 48 h for *Aspergillus* and *Fusarium spp*., and after 72 h for *B. cinerea*, according to the fungal growth control. TDM and CPZ were used as positive controls (12.5–50 μg/mL). Stock solutions were two-fold diluted with RPMI from 1000 to 0.98 μg/mL (final volume equal to 100 μL), and a final DMSO concentration ≤ 1%. An inoculum suspension (100 μL) was added to each well with the exception of the sterility control. Endpoints were defined as the lowest compound concentration resulting in total growth inhibition (MIC_100_) compared to the growth in control wells. MICs values ≤ 50 μg/mL were considered of interest. The assay was carried out in triplicate.

### In vivo antifungal activity

The in vivo activity was determined on the *Vitis vinifera* grapes inoculated with a *Botrytis cinerea* conidia suspension [31]*.* Appropriate amounts of the tested compounds were dissolved in DMSO and then suspended in the distilled water (DMSO concentration < 2%).

Healthy grapes, previously washed with a diluted solution of sodium hypochlorite, were punctured with 100 ⎧L of a solution of compound **4** (concentration of 12.5 ⎧g/mL and 2.5 ⎧g/mL) and evaporated at room temperature (~ 25 °C) during 24 h to favour the penetration of the compound. The same procedure was carried out with CPZ and the control blank (< 2% DMSO solution). The grapes epidermis was punctured with a sterile inoculation needle (Ø, 4.5 mm), 200 µL of the pathogen was inoculated at a concentration of 5.10^4^ UFC/mL. The fruits were incubated at 20 °C to favour fungal growth, and fungal growth was assessed 7 days later. CPZ was used as positive control under the same conditions. The control effectiveness of the target compound was calculated as (1 − *c/d*) × 100, where *c* is the diameter of the treatment and *d* is the diameter of the blank control (˂ 2% DMSO solution).

### Effect on *B. cinerea* hyphal morphology

The effect of the most active compound **4** on the morphology of the hyphae with the mycelia after 7 days of culture of *B. cinerea* treated with the compound was assessed by optical microscopy (40x). The same procedure was carried out with CPZ and blank control (< 2% DMSO solution).

### Cytotoxicity

#### MRC-5 cell culture

The cytotoxic effect of the compounds, expressed as cell viability, was assessed on a permanent fibroblast cell line derived from human lung (MRC-5) (ATCC CCL-171). MRC-5 fibroblasts were grown as monolayers in minimum essential Eagle medium (MEM) with Earle’s salts, 2 mM l-glutamine and 2.2 g/L sodium bicarbonate, supplemented with 10% heat inactivated fetal bovine serum (FBS), 100 IU/mL penicillin and 100 µg/mL streptomycin in a humidified incubator with 5% CO_2_ in the air at 37 °C. Cell passage was maintained between 10 and 16. The medium was changed every 2 days.

### Cytotoxicity assay

Confluent cultures of MRC-5 fibroblasts were treated with medium containing the compounds at concentrations ranging from 0 to 1000 µM. For comparison purposes, the commercial antifungals CPZ and TDM were used as references, the products were first dissolved in DMSO and then in MEM, supplemented with 2% FBS. The final content of DMSO in the test medium and controls was 1%. Cells were exposed for 24 h to the test medium with or without the compound (control). Each concentration was tested in quadruplicate together with the control, and repeated three times in separate experiments. At the end of the incubation, the neutral red uptake (NRU) assay was carried out [32]. To calculate the IC_50_ values (concentration that produces a 50% inhibitory effect on the evaluated parameter), the results were transformed to percentage of controls and the IC_50_ values were graphically obtained from the dose–response curves.

### Lipophilicity

The lipophilicity of the compounds was estimated using the ChemBioDraw Ultra 12.0. The parameter is presented as log P (Table 2).

### Statistical analysis

Results were expressed as the mean ± S.E.M. In all experiments, statistical differences between several treatments and their respective control were determined by one-way analysis of variance (ANOVA), and when the F value was significant, post hoc differences were determined by the Dunnett’s multiple comparison test. The level of significance was set at p < 0.05. All statistical analyses were performed using the software Statistica 5.1 (StatSoft, Inc.).

### Molecular modelling

#### Homology modelling

The amino acid sequence of *B. cinerea* CYP51 (Accession Number: AAF85983) was taken from the NCBI protein database (http://www.ncbi.nlm.nih.gov/ protein). Two crystal structures of *A. fumigatus* CYP51 (PDB codes 4UYL and 4UYM) were used as the crystallographic coordinate templates [18]. The structural model obtained from homology for CYP51 of *B. cinerea* was obtained using Swiss-model [33]. The docking study was carried out using this structural model.

### Molecular docking

Automatic docking was performed using the Autodock Vina 1.1.1. During the docking procedures, water molecules and ligands were removed from the protein. The receptor structure was defined as rigid, and the grid dimensions were 25, 25, and 25 for the X, Y, and Z axes, respectively, taking the active site of the BcCYP51 as the centre of coordinates. Gasteiger charges were assigned to all the compounds, and non-polar hydrogen atoms were merged. All torsions of the ligand were allowed to rotate during docking. The value for the exhaustiveness of the search was 400, whereas the number of poses collected was 10. All graphic manipulations and visualisations were performed by means of the AutoDock Tools 1.5.4 and ligand docking with Autodock Vina 1.1.1.34.

### Molecular dynamics (MD) simulations

The geometries of the complexes obtained from docking were soaked in boxes of explicit water using the TIP3P model and subjected to MD simulation. All MD simulations were performed with the Amber 14 software package using periodic boundary conditions and cubic simulation cells. The particle mesh Ewald method (PME) was applied using a grid spacing of 1.2 Å, a spline interpolation order of 4, and a real space direct sum cut off of 10 Å. The SHAKE algorithm was applied, allowing for an integration time step of 2 fs. MD simulations were carried out at a 310 K temperature. Three MD simulations of 5 ns were conducted for each system under different starting velocity distribution functions; thus, in total, 15 ns were simulated for each complex. The NPT ensemble was employed using Berendsen coupling to a baro/thermostat (target pressure 1 atm, relaxation time 0.1 ps). Post MD analysis was carried out with the program CPPTRAJ.

## Supplementary Information

**Additional file 1:** Histograms of interaction energies partitioned with respect to the amino acids of 14α-demethylase complexed with compounds: **a**) 7 and **b**) 8. The x-axis denotes the residue number of 14α-demethylase, and the y-axis denotes the interaction energy between the compounds and specific residue. Negative values and positive values are favorable or unfavorable to binding, respectively.

## References

[1] Dean R, Van Kan JAL, Pretorius ZA, Hammond-Kosack KE, Di Pietro A, Spanu PD, Rudd JJ, Dickman M, Kahmann R, Ellis J, Foster GD (2012). The top 10 fungal pathogens in molecular plant pathology. Mol Plant Pathol.

[2] Hiner, CC, Townsend, CG, Lavy, BL, Harm J. de Blij’s 1983 Wine: a geographic appreciation. Prog Phys Geog Earth Environ. 2014;38:674-684.

[3] Masih EI, Slezack-Deschaumes S, Marmaras I, Barka EA, Vernet G, Charpentier C, Adholeya A, Paula B (2001). Characterisation of the yeast *Pichia membranifaciens* and its possible use in the biological control of Botrytis cinerea, causing the grey mould disease of grapevine. FEMS Microbiol Lett..

[4] Williamson B, Tudzynsk B, Tudzynski P, van Kan JAL (2007). Botrytis cinerea: the cause of grey mould disease. Mol Plant Pathol.

[5] Droby S, Lichter A, Elad Y, Williamson B, Tudzynski P, Delen N (2007). Post-harvest Botrytis infection: etiology, development and management. Botrytis: biology, pathology and control.

[6] Rupp S, Weber RWS, Rieger D, Detzel P, Hahn M (2017). Spread of *Botrytis cinerea* strains with multiple fungicide resistance in German horticulture. Front Microbiol.

[7] Berthiller F, Crews C, Dall'Asta C, De Saeger S, Haesaert G, Karlovsky P, Oswald IP, Seefelder W, Speijers G, Stroka J (2013). Masked mycotoxins: a review. Mol Nutr Food Res.

[8] Visconti A, Perrone G, Cozzi G, Solfrizzo M. Managing ochratoxin A risk in the grape-wine food chain. Food Add Contam. 2008;25:193–202. 10.1080/02652030701744546 18286409

[9] Lamberth C, Dinges J. The significance of heterocycles for pharmaceuticals and agrochemicals. In: Luzzio FA, editor. Imides: medicinal, agricultural, synthetic applications and natural products chemistry. Wiley-VCH: Weinheim; 2012, p. 3–20.

[10] Lamberth C (2013). Heterocyclic chemistry in crop protection. Pest Manag Sci.

[11] Sheehan DJ, Hitchcock CA, Sibley CM (1999). Current and emerging azole antifungal agents. Clin Microbiol Rev.

[12] Roberts TR, Hutson DH, Lee PW, Nicholls PH, Plimmer JR. Metabolic pathways of agrochemicals, Part 2: insecticides and fungicides. In: Roberts TR, Hutson DH, Lee PW, Nicholls PH, Plimmer JR, eds. RSC: Cambridge. 1999; p. 1011–1101.

[13] Have AT, Dekkers E, Kay J, Phylip LH, van Kan JAL (2004). An aspartic proteinase gene family in the filamentous fungus *Botrytis cinerea* contains members with novel features. Microbiol..

[14] Cilindre C, Jégou S, Hovasse A, Schaeffer C, Castro AJ, Clément C, Van Dorsselae A, Jeandet P, Marchal R (2008). Proteomic approach to identify champagne wine proteins as modified by *Botrytis cinerea* infection. J Proteome Res.

[15] Choi S (2014). Critical review on the carcinogenic potential of pesticides used in Korea, Asian Pac. J Cancer Prev..

[16] Roelofs MJE, Temming RA, Piersma AH, van den Berg M, van Duursen MBM (2014). Corrigendum to “Conazole fungicides inhibit Leydig cell testosterone secretion and androgen receptor activation in vitro. Toxicol Rep.

[17] Heusinkveld HJ, Molendijk J, van den Berg M, Westerin RHS (2013). Azole fungicides disturb intracellular Ca2+ in an additive manner in dopaminergic PC12 cells. Toxicol Sci.

[18] Hargrove TY, Wawrzak Z, Lamb DC, Guengerich FP, Lepesheva GI (2015). Structure-functional characterization of cytochrome P450 sterol 14α-demethylase (CYP51B) from *Aspergillus fumigatus* and molecular basis for the development of antifungal drugs. J Biol Chem.

[19] Becher R, Wirsel SG (2012). Fungal cytochrome P450 sterol 14α-demethylase (CYP51) and azole resistance in plant and human pathogens. Appl Microbiol Biotechnol..

[20] Mellado E, Garcia-Effron G, Buitrago MJ, Alcazar-Fuoli L, Cuenca-Estrella M, Rodriguez-Tudela JL (2005). Targeted gene disruption of the 14-α sterol demethylase (cyp51A) in *Aspergillus fumigatus* and its role in azole drug susceptibility. Antimicrob Agents Chemother.

[21] Herrera Cano N, Santiago AN (2014). Arylation of aryl chlorides, a convenient method for the synthesis of new potential triazolic fungicides. Tetrahedron.

[22] Rossi RA, Griesbeck AG, Mattay J (2005). Photoinduced aromatic nucleophilic substitution reactions in synthetic organic photochemistry. Synthesis organic photochemistry.

[23] Rossi RA, Pierini AB, Santiago AN. Organic Reactions. In: Paquette LA, Bittman R, editors. Wiley: New York; 1999. p. 1–127.

[24] Farina V, Krishnamurthy V, Scott WJ (1997). Stille reaction. Org React.

[25] Basso SM, Montañez JP, Santiago AN (2008). Synthesis and evaluation of phytotoxicity of disugran analogues. Lett Org Chem.

[26] Miyaura NA (1995). Palladium-catalyzed cross-coupling reactions of organoboron compounds. Chem Rev.

[27] Hooshmand S, Heidari B, Sedghi R, Varma RS (2019). Recent advances in the Suzuki-Miyaura cross-coupling reaction using efficient catalysts in eco-friendly media. Green Chem..

[28] Jacobus S, De Angelique R. WO 2012/117051. U.S. Patent 1–42. 2012.

[29] Rosenkranz HS, Matthews EJ, Klopman G (1992). Relationships between cellular toxicity, the maximum tolerated dose, lipophilicity and electrophilicity. ATLA Altern Lab Anim.

[30] Kumar A, Sharma P, Kalal BL, Chandel LK (2010). Synthesis and metal extraction behavior of pyridine and 1, 2, 4-triazole substituted calix [4] arenes. J Incl Phenom Macrocycl Chem.

[31] Rex JH, et al. 27-A3: reference method for broth dilution antifungal suceptibility testing of yeats; approved standard, 3rd edn. CLSI. pp. 1–13; 2008.

[32] Rodríguez JA, Haun M (1999). Cytotoxicity of trans-dehydrocrotonin from *Croton cajucara* (Euphorbiaceae) on V79 cells and rat hepatocytes. Planta Med.

[33] Waterhouse A, Bertoni M, Bienert S, Studer G, Tauriello G, Gumienny R, Heer FT, De Beer TAP, Rempfer C, Bordoli L, Lepore R, Schwede T (2018). SWISS-MODEL: homology modelling of protein structures and complexes. Nucleic Acids Res.

