# Supplementary material for: Arylated analogues of cypronazole: fungicidal effect and activity on human fibroblasts. Docking analysis and molecular dynamics simulations
Source: Nat Prod Bioprospect. 2022 Mar 9;12(1):9. doi: 10.1007/s13659-022-00329-0 (PMC8907375; doi:10.1007/s13659-022-00329-0)

Additional file 1: Figure S1. Histograms of interaction energies partitioned with respect to the amino acids of 14α-demethylasecomplexed with compounds: a) **7** and b) **8**. The x-axis denotes the residue number of 14α-demethylase, and the y-axis denotes the interaction energy between the compounds and specific residue. Negative values and positive values are favorable or unfavorable to binding, respectively.

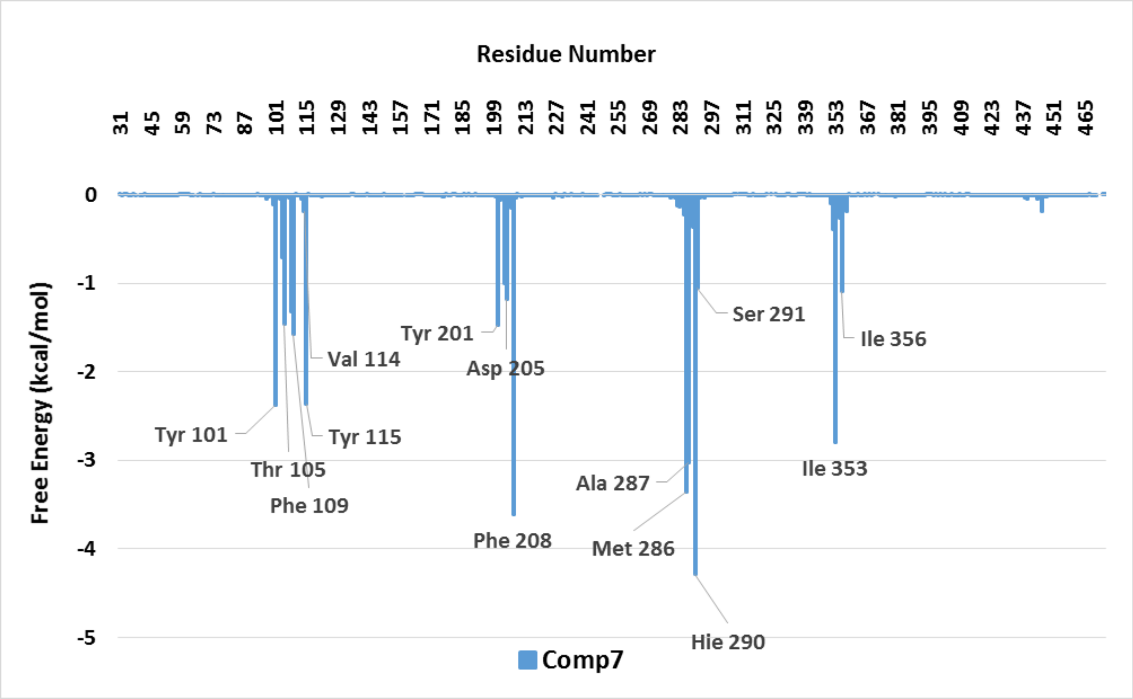

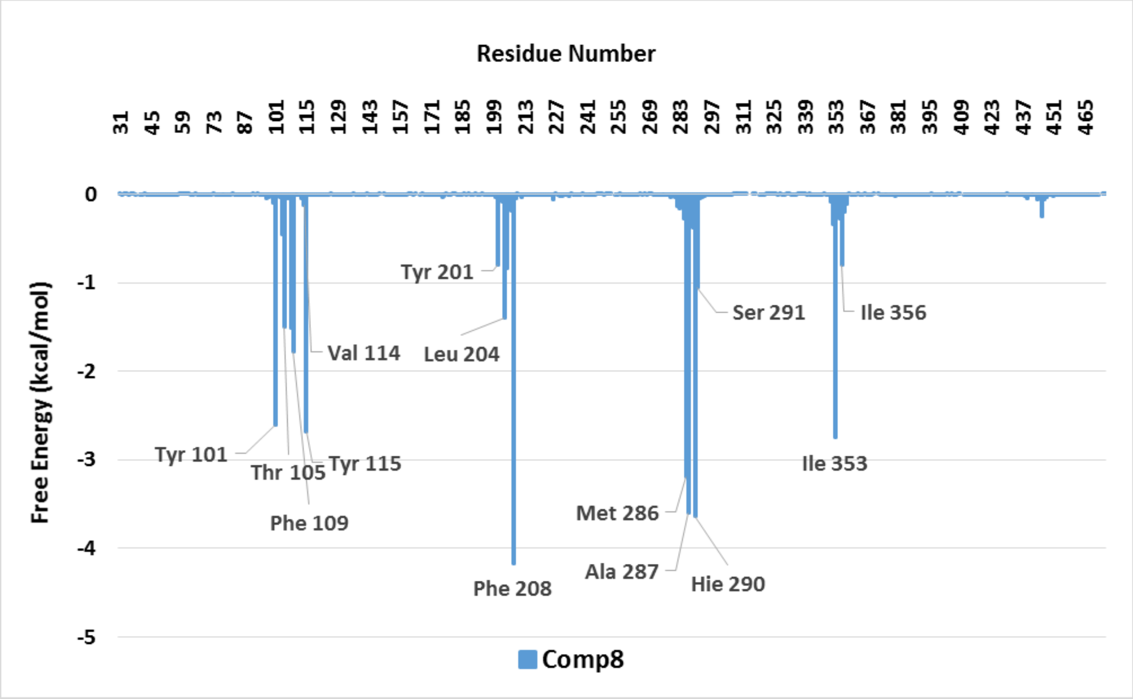

Supplement: Supplementary file 1 — Additional file 1: Histograms of interaction energies partitioned with respect to the amino acids of 14α-demethylase complexed with compounds: a) 7 and b) 8. The x-axis denotes the residue number of 14α-demethylase, and the y-axis denotes the interaction energy between the compounds and specific residue. Negative values and positive values are favorable or unfavorable to binding, respectively. [file 13659_2022_329_MOESM1_ESM.pdf]
